# Fine-Mapping of a Red-Skinned Taproot Gene in Radish (*Raphanus sativus* L.)

**DOI:** 10.3390/plants14193065

**Published:** 2025-10-03

**Authors:** Zhao Liu, Zhenzhen Li, Gaizhen Li, Linyi Qiao

**Affiliations:** 1 College of Horticulture, Shanxi Agricultural University, Taiyuan 030031, China; 2 College of Agronomy, Shanxi Agricultural University, Taiyuan 030031, China

**Keywords:** radish, red skin, bulked-segregant analysis, RNA-sequencing, fine mapping, MYB

## Abstract

The skin color of radish taproots is an important commodity character that directly affects the choice behavior of consumers. Here, we identified a skin color gene carried by a red-skinned inbred line, SXAU-R2. Genetic population was constructed by the crossing of SXAU-R2 and a white-skinned inbred line, SXAU-W2, and the taproots of F_1_ plants exhibited intermediate color. In the F_2_ population, the separation ratio of taproot skin color indicated that the phenotype was controlled by one major locus, named *RST1* (*Red-Skinned Taproot 1*). Combined with bulked segregant analysis and RNA sequencing (BSA-seq), 2640 single nucleotide polymorphisms (SNPs) were detected between the annotated genes of the red skin bulk and white skin bulk. Molecular markers were developed in the SNP-enriched 27~32 Mbp region of chromosome 7, and then *RST1* was mapped in the genetic interval between flanking markers *SSR-14* and *SSR-22*. Using F_2:3_ lines derived from a key F_2_ heterozygote, *RST1* was narrowed down into a 530 Kbp interval. There were 46 expressed annotated genes in the fine-mapping region, and a gene encoding MYB was selected as the candidate of *RST1*. Finally, based on Kyoto Encyclopedia of Genes and Genomes (KEGG) enrichment analysis and RT-qPCR, we identified the potential interacting genes *RsbHLH* and *RsWD*, as well as the latent target genes *RsDFR* and *RsANS* of RST1 in the anthocyanin synthesis pathway. These results provide an understanding of the genetic mechanisms regulating anthocyanin synthesis and offer an efficient molecular marker for the radish breeding of skin color.

## 1. Introduction

Radish (*Raphanus sativus* L., 2n = 2x = 18) is a typical annual or biennial cross-pollinated plant belonging to the Brassicaceae family. It is an important vegetable crop and widely planted in the world, especially in East Asia. Radish commonly accounts for 2% of the total global production of vegetables, and in 2021, the fresh-radish market was worth 1264 million USD [1]. The main edible part of the radish is the fleshy taproot, which is rich in nutrition, high yield, and conducive to storage. In addition to taproot, the leaf, silique, and seed oil of some radish germplasms are also used for food in some areas [2,3].

The taproot of radish displayed abundant diversity in color, shape, size, and flavor, of which the color of taproot skin is an important commodity character of radish that directly affects the choice behavior of consumers. After natural domestication and artificial selection, radish taproots showed different skin colors, including purple, red, pink, white, green, and even black as well as yellow. Among them, red/purple-, green-, and white-skinned radishes are the most common [4]. In a China-specific cultivar (cv.) Xinlimei, the skin color of its taproot is red during the early developmental period and gradually changes to green during maturation. By analyzing the transcriptome data of this process, it was found that the genes involved in anthocyanin biosynthesis were highly up-regulated in the red phase of taproot skin; at the stage when the skin fades red and turns green, anthocyanin-related genes were down-regulated while chlorophyll biosynthesis-related genes were significantly up-regulated [5]. Multiple similar studies confirmed that the skin color of red/purple radish and green radish is formed by the accumulation of anthocyanins and chlorophyll in epidermal cells, respectively [6,7,8].

Several loci for taproot skin color were further identified. Using bulked-segregant analysis (BSA) on an F_2_ population derived from the cross of two inbred lines, green-skinned G-2 and white-skinned W-1, *GREEN-SKINNED TAPROOT 1* (*GST1*) was mapped in the 2.93~8.99 Mbp physical position on the terminal of the short arm of chromosome 1, and annotated genes related to chlorophyll synthesis within this region, such as *RsDnaJ* and *RsPPR*, were highly expressed in the green-skin bulk [9]. Moreover, the red/purple skin of the taproot is mainly regulated by MYB family genes. Lim et al. first isolated *RsMYB1* (GenBank No. KR706195) from red-skinned radish, which is homologous with *PRODUCTION OF ANTHOCYANIN PIGMENT 1* (*AtPAP1*) in Arabidopsis. *RsMYB1* was characterized as a positive regulator to transcriptionally activate the anthocyanin biosynthetic machinery by itself in Arabidopsis and tobacco plants [10]. Based on the F_2_ population crossed by inbred lines CX16Q-25-2 (purple skin) and CX16Q-1-6-2 (white skin), a quantitative trait locus (QTL) for skin color was mapped in the position of 31.58~31.82 Mbp on chromosome 2, and *RsMYB1.1* (gene ID: *Rs094840*) within this region was identified as the candidate gene [11]. Genome-wide association studies on two sets of radish germplasms also detected the QTLs for skin color on chromosome 2, and *RsPAP2* [12] and *RsMYB1.1* (gene ID: *R2.009390*) [13] were selected as the candidate genes, respectively. In addition, on chromosome 7, a skin color QTL was identified in the position of 8.47~12.37 Mbp by using progeny from self-pollination of a Chinese commercial hybrid cv. Lian Yan No. 1 (red skin), and then *RsMYB1* (gene ID: *Rs388430*) in the region was used as the candidate gene [14]. Furthermore, in the F_2_ populations of NAU-YH (red skin) × NAU-LB (white skin), YAAS-RR1 (red skin) × YAAS-WR1 (white skin), and JH6B (red skin) × Minowsh (white skin), skin color QTLs on chromosome 7 were detected, harboring the candidate genes *RsMYB90*, *RsMYB1.3*, and *RsMYB2*, respectively [15,16,17].

SXAU-R2 is a typical red-skinned radish germplasm collected and purified by the Radish Germplasm Resources Innovation and Genetic Breeding Research Group of the College of Horticulture, Shanxi Agricultural University. It is a stable inbred line originated from the Chinese landrace Dahongpao after five generations of self-fertilization. To identify the genetic loci controlling red skin, in this study, a genetic population was constructed using SXAU-R2 and a white-skinned radish, SXAU-W2. Combined with BSA and RNA sequencing (BSA-seq), the gene controlling red skin, *RED-SKINNED TAPROOT 1* (*RST1*), was fine-mapped. These results are beneficial for a deeper understanding of the color mechanism of taproot red skin and provide new molecular markers for quality breeding in radish.

## 2. Results

### 2.1. Genetic Dissection of Radish Skin Color

**Figure 1:**
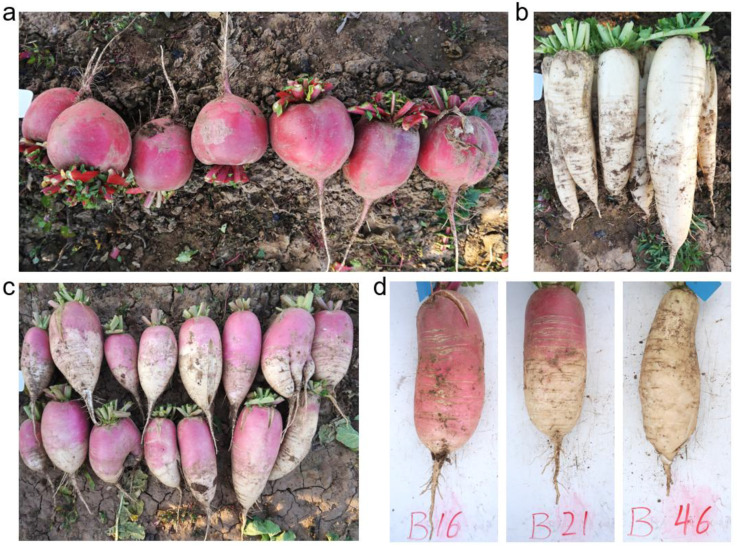
Taproot skin color of two inbred lines, SXAU-R2 and SXAU-W2, as well as their hybrid offspring. (**a**) Red-skinned parent SXAU-R2. (**b**) White-skinned parent SXAU-W2. (**c**) F_1_ generation. (**d**) Red-skinned, intermediate-color-skinned, and white-skinned individuals of the F_2_ segregant population.

**Figure 2:**
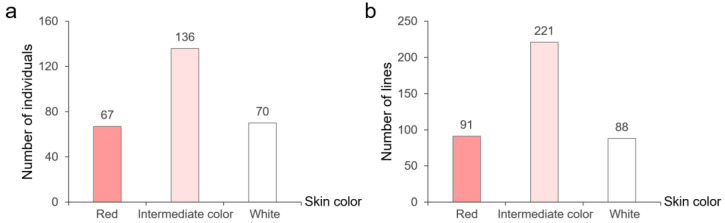
Segregation of taproot skin color in SXAU-R2 × SXAU-W2 F_2_ population (**a**) and F_2:3_ lines derived from a heterozygous F_2_ individual #21 (**b**).

The taproots of radish inbred line SXAU-R2 are spherical and red-skinned (Figure 1a), while those of inbred line SXAU-W2 are cylindrical and white-skinned (Figure 1b). The hybrid F_1_ plants of parents SXAU-R2 and SXAU-W2 displayed an intermediate phenotype of root skin color, with red near the leaves and white at the bottom (Figure 1c). In the SXAU-R2 × SXAU-W2 F_2_ population, three types of root skin color were separated, including red, intermediate color, and white (Figure 1d). The statistical results showed that there were 67 red-skinned individuals, 136 intermediate-skinned individuals, and 70 white-skinned individuals (Figure 2a), with a ratio roughly in line with 1:2:1 (*χ*^2^ = 0.97, *p* > 0.05), indicating that the root skin color was controlled by one major locus, which is temporarily named *RST1* (*Red-Skinned Taproot 1*).

### 2.2. Distribution of Bulk-Polymorphic SNPs

**Figure 3:**
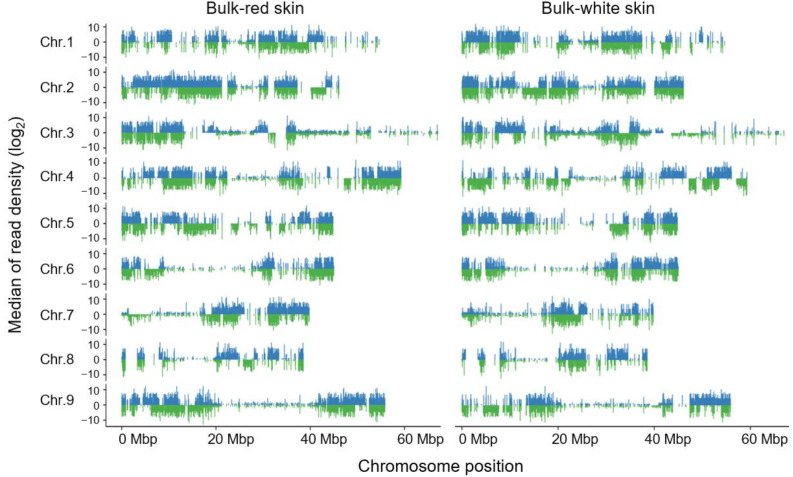
Genome-wide distribution of read coverage of the BSA-seq data. Taking 10 Kbp as the interval unit length, the chromosome was divided into multiple small windows, and the mapped reads falling in each window were counted as the coverage depth. Blue represented forward sequence, while green represented reverse sequence.

Using 20 red-skinned and 20 white-skinned individuals of the SXAU-R2 × SXAU-W2 F_2_ population, the red-skin bulk and white-skin bulk were constructed for BSA-seq, respectively. Each bulk obtained clean data above 20 Mb with Q30-based percentages greater than 93.93% and GC percentages ranging from 47.74 to 48.01% (Table S1), indicating that the sequencing data was of high quality for further analysis. The clean reads were then mapped on the radish reference genome (*R. sativus* var. *radicula*) and covered all the nine chromosomes of radish (Figure 3). Subsequently, the uni-transcripts were annotated as genes.

**Figure 4:**
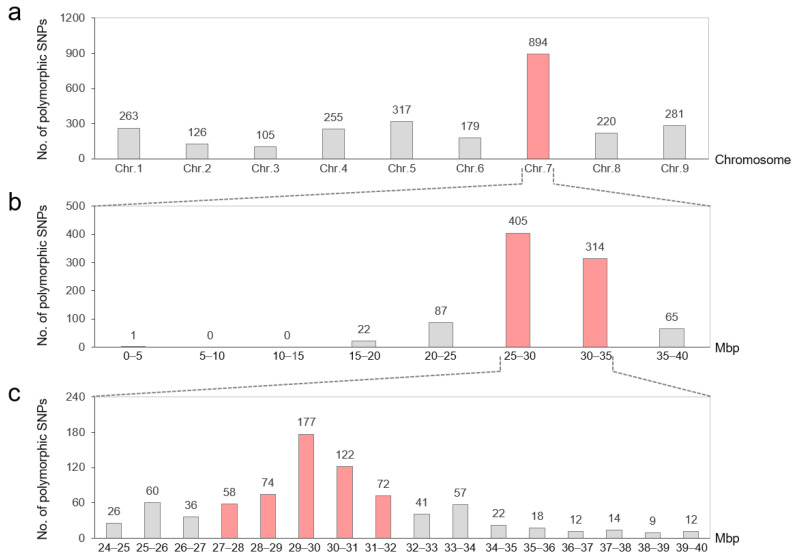
Chromosomal distribution of 2640 SNPs between annotated genes in the red skin bulk and white skin bulk. (**a**) Chromosomes 1~9 of radish. (**b**) Physical position of chromosomes 7. (**c**) The position of 28~32 Mbp on chromosome 7. The chromosome or physical position with high SNP distribution was marked in red.

Based on sequence alignment, 2640 SNPs were detected between annotated genes in the red skin bulk and white skin bulk, with the distribution numbers on chromosomes 1 to 9 being 263, 126, 105, 255, 317, 179, 894, 220, and 281, respectively (Figure 4a, Table S2). Among them, the most SNPs were found on chromosome 7 (33.86%), mainly concentrated in the physical position of 27~32 Mbp (Figure 4b,c), indicating that *GST1* is probably located on chromosome 7. It is worth noting that the SNPs were also widely distributed on other chromosomes, ranging in number from 105 to 317, which may be due to high background noise caused by the small bulk size.

### 2.3. Initial Mapping of RST1

**Figure 5:**
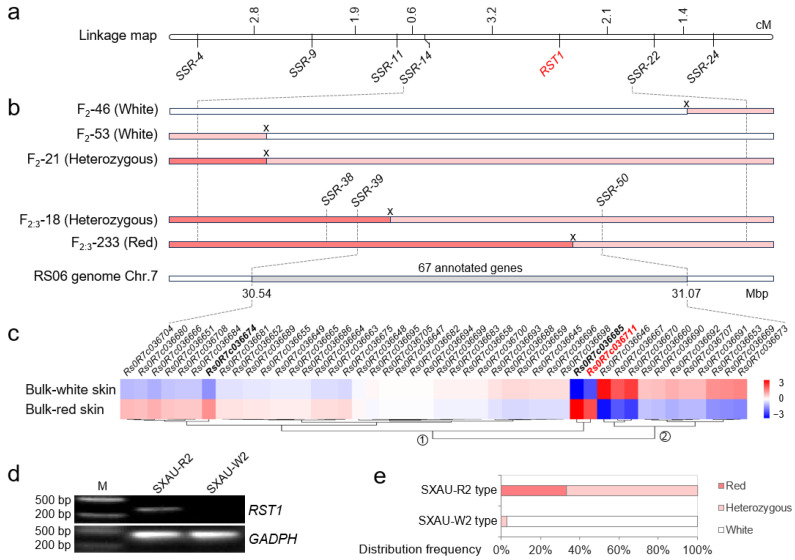
Fine mapping of *RST1*. (**a**) Linkage map of *RST1* in the SXAU-R2 × SXAU-W2 F_2_ population. *RST1* was marked in red. (**b**) Diagrams of the locations of crossovers in three recombinant F_2_ individuals and two F_2:3_ lines deriving from the F_2_-21 individual. The red box referred to the SXAU-R2 allele, the white box referred to the SXAU-W2 allele, pink box referred to the heterozygote, and the gray box referred to the targeted region of *RST1*. ‘X’ indicated a crossover event. (**c**) Heatmap of the expressed annotated genes within the *RST1* region based on the BSA-seq data. Genes significantly up-expressed in the red-skinned bulk were highlighted in bold, and the candidate gene was marked in red. (**d**) Agarose gel electrophoresis results of the diagnostic marker developed for the causing gene *Rs0R7c036711* of *RST1*. The radish *GADPH* gene was used as the positive control. The diagnostic marker of RST1 had an amplification product in red-skinned parent SXAU-R2, but not in white-skinned parent SXAU-W2. Marker of positive control GADPH had amplification products in both parents. M: DNA ladder marker. (**e**) Phenotype of taproot skin color of F_2_ individuals corresponding to the two alleles of *RST1* that were detected by the diagnostic marker.

Based on simple sequence repeat (SSR) loci in the SNP-enriched 27~32 Mbp region of chromosome 7, in total 30 molecular markers (*SSR1~SSR30*) were randomly developed and used to amplify parents SXAU-R2 and SXAU-W2. Then, six parental polymorphic markers, including *SSR-4*, *SSR-9*, *SSR-11*, *SSR-14*, *SSR-22*, and *SSR-24*, were selected to amplify the F_2_ population. Combined with phenotypes of root skin color, *RST1* was mapped between *SSR14* and *SSR22*, with genetic distances of 3.2 cM and 2.1 cM, respectively, corresponding to the physical position of 29.70~31.64 Mbp on chromosome 7 (Figure 5a).

### 2.4. Fine-Mapping of RST1

One key heterozygous F_2_ individual, #21, was selected for fine-mapping (Figure 1d). The F_2_-21 plant harvested a total of 923 seeds, and 400 seeds were randomly selected for planting and generating F_2:3_ plants. Results of phenotype identification showed that the number of F_2:3_ plants with red-skinned, intermediate-skinned, and white-skinned roots were 91, 221, and 88, respectively, roughly in a 1:2:1 ratio (*χ*^2^ = 0.11, *p* > 0.05) (Figure 2b), once again confirming that the root skin color is controlled by the major gene *RST1*. Twenty SSR markers, *SSR31~SSR50*, were further developed in the initial mapping region of *RST1* (29.70–31.64 Mbp), of which three markers showed parental polymorphism and were then used to amplify the F_2:3_ plants deriving from F_2_-21. Luckily, new genetic exchange events were detected in two plants, F_2:3_-18 and F_2:3_-233, narrowing down *RST1* into the physical position of 30.54~31.07 Mbp between markers *SSR39* and *SSR50* (Figure 5b).

### 2.5. Determination of Candidate Gene

There were 67 annotated genes within the fine-mapped region of *RST1*, only 46 of which were expressed in the root epidermis, according to the BSA-seq results. Based on expression patterns, these 46 expressed genes were divided into two categories: 11 genes up-regulated in the white skin bulk and 35 genes up-regulated in the red skin bulk (Figure 5c). Among them, three genes were significantly upregulated in the red skin bulk (|log2FoldChange| ≥ 1 and FDR ≤ 0.01), including *Rs0R7c036674*, *Rs0R7c036685*, and *Rs0R7c036711*, which encoded E3 ubiquitin protein ligase, alcohol dehydrogenase class-P, and MYB transcription factor, respectively (Table S3).

As several R2R3-type MYBs characterized by N-terminal R2 and R3 DNA-binding domains have been reported to regulate pigment synthesis in plants [18], *Rs0R7c036711* was selected as the candidate gene of *RST1*. We developed a diagnostic marker for *Rs0R7c036711*, which had amplification product in the red-skinned parent but no amplification product in the white-skinned parent (Figure 5d). The genotyping results in the F_2_ population showed that the individuals with the SXAU-R2 allele all exhibited red-skinned or intermediate-skinned roots, while the individuals with the SXAU-W2 allele basically displayed white-skinned roots (97.22%) (Figure 5e), indicating that this marker is co-segregated with the skin color phenotype and confirming that *Rs0R7c036711* is the causing gene.

### 2.6. Expression Patterns of Genes Involving Anthocyanin Synthesis

**Figure 6:**
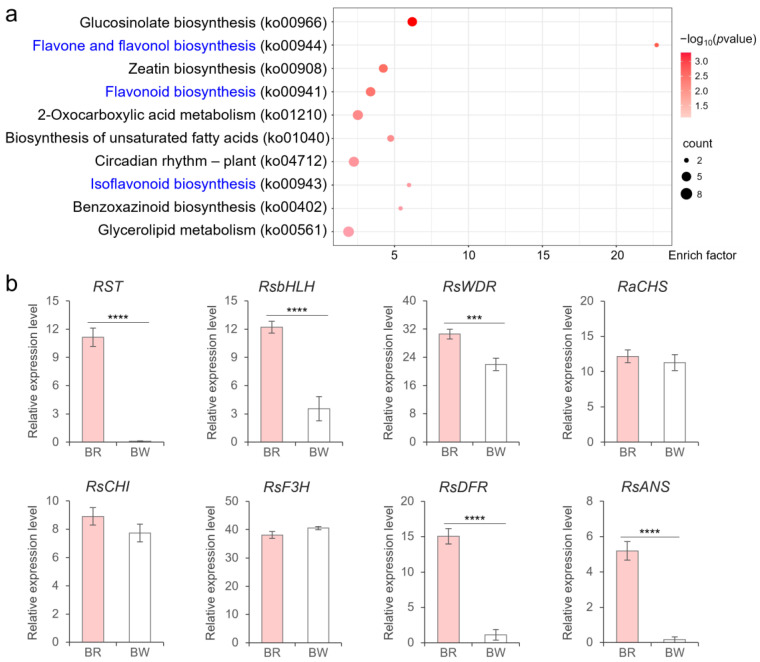
DEGs between red-skinned bulk and white-skinned bulk. (**a**) The top 10 terms of KEGG enrichment results for DEGs. Term names related to flavonoid biosynthesis were marked in blue. The dot color and the dot size indicated the significance of enrichment and the count of enriched genes in terms, respectively. (**b**) Relative expression level of *RST1*, *RsbHLH*, *RsWDR*, and genes involved in anthocyanin synthesis. CHS: chalcone synthase; CHI: chalcone isomerase; F3H: flavanone 3-hydroxylase; DFR: dihydroflavonol-4-reductase; ANS: anthocyanidin synthase. BR: red-skinned bulk; WR: white-skinned bulk. *** indicates *p* < 0.001, and **** indicates *p* < 0.0001.

To speculate on the possible regulatory mechanism of RST1 on skin color, we conducted Kyoto Encyclopedia of Genes and Genomes (KEGG) enrichment analysis on differentially expressed genes (DEGs) between the red skin bulk and white skin bulk. The results showed that three of the top 10 KEGG terms were related to anthocyanin synthesis, namely flavone and flavonol biosynthesis (ko00944), flavonoid biosynthesis (ko00941), and isoflavonoid biosynthesis (ko00943) (Figure 6a, Table S4). Therefore, we inferred that *RST1* was involved in regulating the process of flavonoid synthesis.

From the BSA-seq data, we isolated the *BASIC HELIX-LOOP-HELIX* gene *RsbHLH* (*Rs0R9c043795*) and the *WD40 REPEAT* gene *RsWDR* (*Rs0R6c033946*), as well as genes encoding for key enzymes in the anthocyanin synthesis pathway, including *CHALCONE SYNTHASE* (*RsCHS*), *CHALCONE ISOMERASE* (*RsCHI*), *FLAVANONE 3-HYDROXYLASE* (*RsF3H*), *DIHYDROflAVONOL-4-REDUCTASE* (*RsDFR*), and *ANTHOCYANIDIN SYNTHASE* (*RsANS*). RT-qPCR results showed that the expression levels of *RST1*, *RsbHLH*, and *RsWDR* were significantly higher in the red skin bulk than in the white skin bulk (Figure 6b). Moreover, *RsDFR* and *RsANS* were also highly expressed in the red skin bulk, suggesting that RST1 may co-regulate the transcription of *RsDFR* and *RsANS* with RsbHLH and RsWDR (Figure 6b).

## 3. Discussion

### 3.1. RST1 Can Be Used for the Radish Breeding of Skin Color

Radish is an important root vegetable grown and consumed throughout the world. With the improvement of people’s consumption level, red/purple radishes have become increasingly popular due to their enriched glucosinolates and anthocyanins [19]. As a meaningful flavonoid compound, anthocyanins possess potent antioxidant capacity that is beneficial for health, including protecting against diabetes and cardiovascular diseases [20]. Moreover, anthocyanins play key roles in plants for attracting pollinators and seed dispersers, as well as protecting high light stress and pathogen attack [21]. Therefore, breeding radish cultivars with red/purple skin has become an objective with market value. Marker-assisted selection (MAS) can greatly accelerate the breeding process. In the past decade, several radish genetic maps have been developed based on traditional molecular markers [22,23,24]. However, these maps with low-density markers are still not effective for fine mapping of genes or QTLs. The insufficiency of effective markers that are closely linked with target genes limited the application of MAS in radish breeding. Recently, some costly biological techniques, such as whole-genome sequencing (WG-seq), or specific-locus amplified fragment sequencing (SLAF-seq) have been used to construct the high-density genetic maps of radish [15,16]. In our study, using the low-cost but high-accuracy BSA-seq, *RST1* was successfully fine-mapped in a 530 Kbp interval. Due to the presence of a highly probable candidate gene, *MYB*, within this region, further high-resolution mapping was not performed. Finally, a functional marker of *RST1* was developed and co-separated with the phenotype, providing an efficient molecular marker for the radish breeding of skin color. The enrichment of anthocyanins in taproot epidermis can enhance the tolerance of radish to pathogens or abiotic stresses and contribute to sustainable agricultural production.

### 3.2. RST1 Participates in Regulating Anthocyanin Synthesis

**Figure 7:**
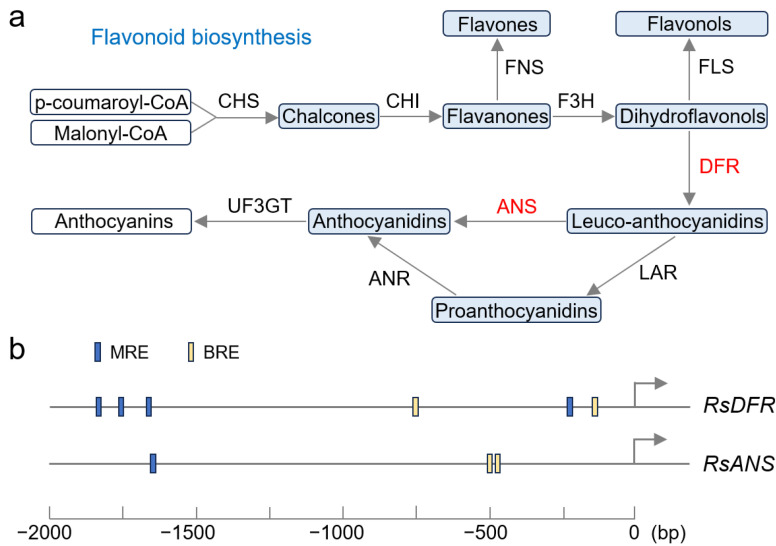
The flavonoid biosynthesis pathway. (**a**) Biochemical reaction steps of flavonoid synthesis. Chemical products were shown in boxes, and enzymes were shown next to the arrows. The enzymes marked in red indicated that its coding gene was highly expressed in the red-skinned bulk in this study. CHS: chalcone synthase; CHI: chalcone isomerase; FNS: flavone synthase; F3H: flavanone 3-hydroxylase; FLS: flavonol synthase; DFR: dihydroflavonol-4-reductase; ANS: anthocyanidin synthase; LAR: leucoanthocyanidin reductase; ANR: anthocyanidin reductase; UF3GT: flavonoid 3-O-glucosyltransferase. (**b**) Putative binding sites of the MBW complex in the promoter region of *RsDFR* (*Rs0R9c041942*) and *RsANS* (*Rs0R4c020786*). The blue and yellow small rectangles represented the MYB-recognizing element (MRE) and bHLH-recognizing element (BRE), respectively.

We identified an *MYB* gene as the candidate for *RST1*. In Arabidopsis, MYB can bind with bHLH transcription factor and WDR protein to form a MYB-bHLH-WDR (MBW) complex that regulates anthocyanin synthesis [25]. Among them, MYB was the core of the complex and was responsible for recognizing and binding to the specific site of the target gene promoter; bHLH showed a weak ability to bind to DNA, but it strongly combined with MYB and helped the complex to recruit other regulatory factors, while WDR acted as a scaffold to stabilize the whole ternary complex [25,26,27]. In this study, the expression levels of *RST1*, *RsbHLH*, and *RsWDR*, as well as *RsDFR* and *RsANS* in the red skin bulk, were significantly higher than those in the white skin bulk, suggesting that the RST1-RsbHLH-RsWDR complex may regulate the reaction steps from dihydroflavonols to anthocyanidins in the anthocyanin synthesis pathway (Figure 7a) [28]. MYB and bHLH of the complex could recognize and bind specific elements in the promoter region of *DFR* and *ANS* [12,29]. These reported binding sites were also identified in the promoter of *RsDFR* (*Rs0R9c041942*) and *RsANS* (*Rs0R4c020786*) in our study (Figure 7b), and we will verify them through electrophoretic mobility shift assays or luciferase assays in the future.

### 3.3. Relationship Between RST1 and Anthocyanin-Related MYBs

**Figure 8:**
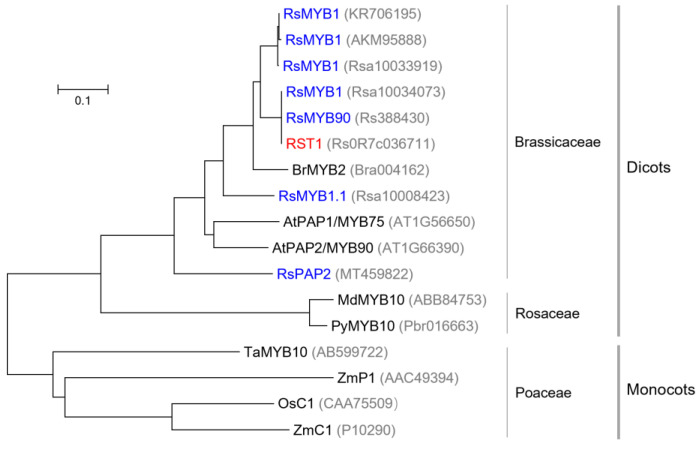
Phylogenetic analysis of flavonoid-related MYBs based on their amino acid sequences. The accession numbers of the MYBs were listed in brackets. MYBs in radish were marked in blue, and the MYB encoded by *RST1* in this study was marked in red. In addition, AtPAP1/MYB75 and AtPAP2/MYB90 were from *Arabidopsis thaliana*, BrMYB2 from Chinese cabbage (*Brassica rapa*), MdMYB10 from apple (*Malus domestica*), PyMYB10 from pear (*Pyrus*), TaMYB10 from wheat (*Triticum aestivum*), ZmP1 and ZmC1 from maize (*Zea mays*), and OsC1 from rice (*Oryza sativa*). Bar meant 0.1 substitutions per site.

At present, several *MYB* genes controlling red or purple radish root skin have been identified based on numerous versions of the radish genome [30,31,32]. Compared with the above research, we further revealed that MYB may control red color by regulating the transcription levels of anthocyanin pathway genes *RsDFR* and *RsANS*. However, due to the lack of a well-established genetic transformation system in radishes, we were still unable to perform functional validation experiments on *RST1*. In addition, the relationship between these *MYBs* is not clear due to the differences in reference genome and naming methods. Some genes are different but share one name, such as *RsMYB1* (*Rsa10034073*) [14] and *RsMYB1* (*Rsa10033919*) [17], while some genes are the same sequence but with different names, such as *RsMYB90* (*Rs388430*) [15] and *RsMYB1a* (*Rs388430*) [33]. Therefore, we constructed a phylogenetic tree for the published *MYB* genes controlling root skin color in radish and several *MYB* genes involved in anthocyanin synthesis in other species (Figure 8). The results showed that *RST1* was the same gene as *RsMYB1* carried by red-skinned cv. Lian Yan No.1 [14] and *RsMYB90* carried by red-skinned inbred line NAU-YH [15]. Moreover, three genes named *RaMYB1* that were detected in different red-fleshed radish germplasm, including *RsMYB1* (KR706195) [34], *RsMYB1* (AKM95888) [10], and *RsMYB1* (*Rsa10033919*) [17], shared the same gene sequence. In addition, MYBs from Brassicaceae, such as radish MYBs, Arabidopsis MYBs, and a Chinese cabbage MYB [35], were clustered into one group; MYBs from Rosaceae, including MdMYB10 from apple and PyMYB10 from pear, were clustered into the second group; while MYBs in gramineous crops (wheat, maize, and rice) from Poaceae were clustered into another group. Overall, MYBs from dicots and MYBs from monocots did not cluster into one group, suggesting that the MYB family differentiated at least after the divergence of monocots and dicots (~130 MYA) [36].

## 4. Material and Methods

### 4.1. Plant Materials

**Figure 9:**
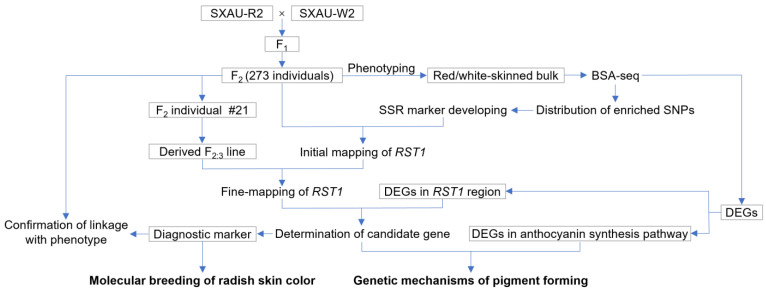
The workflow diagram of this study.

The red-skinned radish SXAU-R2 is an inbred line that originated from the landrace Dahongpao after five generations of self-fertilization, and SXAU-W2 is a white-skinned inbred line selected from the hybrid progeny of landraces Zhentoubai and Shibai, with the characteristics of heat tolerance, disease resistance, and great adaptability. The biparental populations of SXAU-R2 and SXAU-W2, including the F_1_ heterozygote, F_2_ plants with 273 individuals, and F_2:3_ families derived from certain F_2_ plants, were used to identify the red-skinned taproot gene carried by SXAU-R2. The above materials were collected and preserved by the Radish Germplasm Resources Innovation and Genetic Breeding Research Group of the College of Horticulture, Shanxi Agricultural University. The experiments conducted on these materials in this study are shown in Figure 9.

### 4.2. Experimental Conditions and Phenotyping

Seeds were sown in 72-cell plug trays and germinated in a growth chamber set at 22 °C under a 16 h light/8 h dark cycle and 60% humidity. After 1 week of germination, seedlings were transferred to the ridges of hilled rows (0.2 m width and 0.3 m height; 0.3 m separation of rows and columns) of a 2 m × 20 m plot in the experimental field of Shanxi Agricultural University (37°33′ N, 112°40′ E, Jinzhong, Shanxi, China). During the late stage of taproot enlargement (about 75 days after planting), the skin color was investigated visually and recorded as A for red, B for white, and M for intermediate color, based on the parental phenotypes. After phenotyping, genomic DNA was extracted from the tender leaf of each plant using the CTAB method [37].

### 4.3. BSA-Seq

According to the identification results for skin color of the SXAU-R2 × SXAU-W2 F_2_ population, 20 red-skinned individuals were selected, and the taproot epidermis of each individual was sampled and then mixed with equal mass to form a red skin bulk. Using the same method, 20 white-skinned individuals were selected to construct the white skin bulk. These two sample bulks were used to extract total RNA by TRIzol reagent (Invitrogen, Carlsbad, CA, USA) and then construct cDNA libraries using the TruSeq RNA Sample Preparation Kit v2 (Illumina, San Diego, CA, USA) for sequencing on the Illumina NovaSeq6000 platform (Biomarker Tech., Beijing, China). Clean reads were obtained by removing low-quality reads containing adapters and poly-N (>10%) or with a quality score < 30 from the raw data and then mapping them to the radish reference genome (*R. sativus* var. *radicula*) that downloaded from the China National Genomics Data Center (NGDC) database with accession number GWHANWP00000000 (https://ngdc.cncb.ac.cn/, accessed on 20 May 2024).

### 4.4. Development of PCR-Based Markers

Through comparing the assembled gene sequences from the red- and white-skin bulks on the BioMarker Cloud Server (https://international.biocloud.net/, accessed on 1 June 2024), SNPs with information on chromosomal position between bulks were obtained. Next, sequences of genomic segments with high SNP distribution frequency were extracted from the radish reference genome mentioned above and then used for primer design. Briefly, the genome sequence was input into the SSRHunter software (version 1.3) to search for SSR sites, setting the parameters as follows: the nucleotides per repeat unit were two to five, and the repeat times were ≥5. Then, the eligible SSR loci within the target sequence were obtained. In the upstream and downstream 150 bp sequences of each locus, forward and reverse primers were designed, respectively, with the following criteria: primer length = 20 ± 2 bp, theoretical product size < 200 bp, and the annealing temperature difference between the forward and reverse primers was less than 2 °C (Table S5). Polymorphic SSR markers were subsequently screened by genomic DNA of parents SXAU-R2 and SXAU-W2. The amplification condition was as follows: 94 °C for 30 s, 36 cycles of 94 °C for 30 s, 58 °C for 30 s, and then 72 °C for 30 s, with a final extension of 72 °C for 5 min. Polyacrylamide gel electrophoresis was used for differentiating the PCR products. In addition, a diagnostic marker was developed for distinguishing the allelic variations in the causing gene of *RST1* using agarose gel electrophoresis.

### 4.5. Mapping and Fine Mapping

Briefly, the parental polymorphic SSR markers were used to amplify the genomic DNA of the SXAU-R2 × SXAU-W2 F_2_ population. The genotypes, together with the phenotypic data of skin color, were imported into Joinmap software (version 4.0) to calculate the genetic distances between markers and the target gene by using the Kosambi function so as to complete the preliminary mapping of *RST*. For the next fine mapping, the heterozygous F_2_ individuals were selected by flanking markers of *RST*, and their seeds were planted to obtain the corresponding F_2:3_ plants. Markers in the initial *RST* section were continuously developed for amplifying the F_2:3_ plants to obtain new genetic exchange events. Combined with the skin color of F_2:3_ plants, the *RST* section was narrowed down.

### 4.6. Transcription Levels of Annotated Genes

Referring to the previous method [38], the transcript level of each gene from the BSA-seq data was measured with fragments per kilobase of exon model per million mapped fragments (FPKM) values calculated from the following formula: FPKM = cDNA Fragments/Mapped Fragments (Millions) × Transcript Length (kb), where “cDNA Fragments” represents the number of fragments mapped to a certain transcript, “Mapped Fragments (Millions)” represents the total number of fragments mapped to the transcript, measured in units of 10^6^, and “Transcript Length (kb)” represents the length of the certain transcript, measured in units of 10^3^ bases. Differentially expressed genes (DEGs) analysis between red skin bulk and white skin bulk was performed using the DESeq package (version 2.0), with a threshold of |log2FoldChange| ≥ 1 and FDR ≤ 0.01. The DEGs were then used for the enrichment analysis of Kyoto Encyclopedia of Genes and Genomes (KEGG). Moreover, the expression levels of annotated genes within the *RST* section in the two bulks were visualized in a heatmap based on their FPKM data using MeV software (version 4.9).

### 4.7. RT-qPCR

The genes encoding key enzymes in the anthocyanin biosynthesis pathway were selected from the BSA-seq data and used to identify the expression levels. As described previously [39], the backup total RNA for BSA-seq was reverse-transcribed into cDNA using a reverse transcription kit (Takara Bio, Shiga, Japan). RT-qPCR was performed on the QuantStudio 3 Real-time PCR System (Applied Biosystems, Carlsbad, CA, USA) utilizing Premix Ex Taq II enzyme (Takara Bio, Shiga, Japan) and specific primer pairs for the anthocyanin biosynthesis-related genes (Table S5), and the radish *GADPH* gene was used as the internal reference gene. Each reaction was repeated three times, and the results were analyzed using the 2^−∆*CT*^ method, wherein the ∆*CT* was calculated using the following formula: ∆*CT* = *CT* (target gene) − *CT* (reference gene).

### 4.8. Statistical Analysis

The Origin (version 3.1) software was used to perform the statistical analysis by one-way analysis of variance (ANOVA), and *p* < 0.05 was considered a significant difference, while *p* < 0.01 was considered an extremely significant difference.

## 5. Conclusions

The red-skinned radish inbred line SXAU-R2 carried a major skin color gene, *RST1*, which was fine-mapped in the physical position of 30.54~31.07 Mbp on chromosome 7. Within the target region, a MYB transcription factor-encoding gene was selected as the candidate gene of *RST1*, and its functional marker was developed. Finally, the potential interacting genes and the latent target genes of RST1 were identified. Our results provide a better understanding of the color mechanism of taproot red skin and offer an efficient molecular marker for quality breeding in radish.

## Data Availability

Data are contained within the article and Supplementary Materials.

## Supplementary Materials

The following supporting information can be downloaded at: https://www.mdpi.com/article/10.3390/plants14193065/s1, Table S1: Summary of BSA-seq results; Table S2: 2640 SNPs between annotated genes in the red skin bulk and white skin bulk; Table S3: 67 annotated genes within the fine-mapped region of RST1; Table S4: Results of KEGG enrichment analysis on DEGs between the red skin bulk and white skin bulk; Table S5: Primers used in this study.
